# Allometric Convergence in Savanna Trees and Implications for the Use of Plant Scaling Models in Variable Ecosystems

**DOI:** 10.1371/journal.pone.0058241

**Published:** 2013-03-06

**Authors:** Andrew T. Tredennick, Lisa Patrick Bentley, Niall P. Hanan

**Affiliations:** 1 Natural Resource Ecology Laboratory and Graduate Degree Program in Ecology, Colorado State University, Fort Collins, Colorado, United States of America; 2 Department of Ecology and Evolutionary Biology, University of Arizona, Tucson, Arizona, United States of America; 3 Geographic Information Science Center of Excellence, South Dakota State University, Brookings, South Dakota, United States of America; Utah State University, United States of America

## Abstract

Theoretical models of allometric scaling provide frameworks for understanding and predicting how and why the morphology and function of organisms vary with scale. It remains unclear, however, if the predictions of ‘universal’ scaling models for vascular plants hold across diverse species in variable environments. Phenomena such as competition and disturbance may drive allometric scaling relationships away from theoretical predictions based on an optimized tree. Here, we use a hierarchical Bayesian approach to calculate tree-specific, species-specific, and ‘global’ (i.e. interspecific) scaling exponents for several allometric relationships using tree- and branch-level data harvested from three savanna sites across a rainfall gradient in Mali, West Africa. We use these exponents to provide a rigorous test of three plant scaling models (Metabolic Scaling Theory (MST), Geometric Similarity, and Stress Similarity) in savanna systems. For the allometric relationships we evaluated (diameter vs. length, aboveground mass, stem mass, and leaf mass) the empirically calculated exponents broadly overlapped among species from diverse environments, except for the scaling exponents for length, which increased with tree cover and density. When we compare empirical scaling exponents to the theoretical predictions from the three models we find MST predictions are most consistent with our observed allometries. In those situations where observations are inconsistent with MST we find that departure from theory corresponds with expected tradeoffs related to disturbance and competitive interactions. We hypothesize savanna trees have greater length-scaling exponents than predicted by MST due to an evolutionary tradeoff between fire escape and optimization of mechanical stability and internal resource transport. Future research on the drivers of systematic allometric variation could reconcile the differences between observed scaling relationships in variable ecosystems and those predicted by ideal models such as MST.

## Introduction

One of the central goals of ecology is to identify and understand the underlying rules and mechanisms that govern the form and function of organisms. In particular, the existence of consistent allometric relationships across diverse taxa has led to theories that attempt to use physical first principles to model biological scaling. For plants, there are several ‘universal’ scaling theories that produce testable predictions including Metabolic Scaling Theory (MST; [1]), the Geometric Similarity model (GEOM; [2]), and the Stress Similarity model (STRESS; [3]) (Table 1). These models all assume physical constraints to arrive at predictions of allometric scaling. However, given the variability inherent in many ecological systems, the utility of these idealized (i.e. “optimal”) models to predict real ecological phenomena [4], [5] across multiple scales of inquiry [6] has come into question [5], [7]–[10] (but see [11], [12]).

**Table 1:** Model predictions for scaling exponents (*b*).

Scaling Model	Length	Aboveground Mass	Stem Mass	Leaf Mass
Metabolic Scaling Theory (MST)	2/3	8/3	8/3	2
Stress Similarity (STRESS)	1/2	5/2	NA	NA
Geometric Similarity (GEOM)	1	3	3	2

The scaling exponents all refer to *b* in Equation 1 where the dependent variable (*X*) is diameter. For example, the 2/3 in upper-left cell shows that under Metabolic Scaling Theory length is proportional to diameter to the 2/3 power (*l
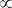
D^2/3^*).

Indeed, the extent to which variability and disturbances such as herbivory and fire may invalidate the allometric predictions of universal models based only on physical first principles remains uncertain. Since these models are based on optimizing assumptions about mechanical constraints that ignore the role of resources (GEOM and STRESS), or optimize resource distribution and plant uptake (MST) they may fail to predict scaling relationships in temporally and spatially heterogeneous environments where resource uptake is constrained by resource limitation [5]. Further, demographic processes may not be entirely resource-based in variable environments where populations may be maintained in a non-equilibrium or disequilibrium state [13], [14] by disturbances and resource pulses [15]. In these cases, selection for traits adaptive under conditions of spatiotemporal variability and disturbance may be more important than selection for optimal mechanical or physiological architecture [16] – the only selective forces invoked by zero-order scaling models (see *Materials and Methods: Scaling models*).

Savannas therefore offer an interesting test case for universal scaling models because the dominant paradigms of savanna ecology invoke competition, environmental variability, and anthropogenic disturbances as mediators of tree cover and structure [17]. Savannas are highly variable two-layer tree-grass systems broadly defined by a discontinuous and dynamic tree layer with a continuous herbaceous layer [18]. Climate plays an integral role in constraining potential tree cover of savannas, but realized tree cover is highly variable in space and time [14]. Moreover, tree biomass and architecture may vary in savannas based on the magnitude and extent of disturbances such as browsing [9] and fire [19]. Inter- and intra-annual variability in precipitation, competition for water, and multiple disturbances including fire, herbivory, and tree harvest establish broad environmental gradients and create conditions that may select for modified allometries and lead to greater allometric variation at the level of individuals and species.

To test the ability of universal scaling models (MST, GEOM, and STRESS) to predict whole-tree and within-tree allometric relationships in variable systems we examine allometric scaling relationships for three tree species from three savanna sites in West Africa. We use a hierarchical Bayesian (HB) approach to estimate scaling parameters (*a*, the normalizing constant, and *b*, the scaling exponent) from the general allometric equation,
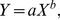
(1)a power-law. In this analysis we treat branch (or basal) diameter as the independent variable (*X*) and calculate its relationship with four branch (or tree) traits: 1) length, 2) aboveground mass, 3) stem mass, and 4) leaf mass (*Y*s in Equation 1). We evaluate the competing scaling models by comparing our empirical estimates to theoretical predictions. All are power-law models that make specific predictions for the scaling exponent *b* (Equation 1) relating plant morphology (*Y*, e.g. length, mass) to plant size (*X*, e.g. diameter).

Specifically, the objectives of our study are to determine: 1) if tree species in savannas exhibit similar scaling relationships for length, aboveground mass, stem mass, and leaf mass; 2) if there is more variability in scaling relationships among or within species; and 3) if the scaling exponents derived from our combined branch and tree data support or reject MST and/or other scaling model predictions. Our main hypothesis is that since universal scaling models make idealizing assumptions regarding plant architecture and the environment within which plants live, we will observe deviations from model predictions for an idealized network structure since savanna trees must respond to variable environmental conditions. To assess this hypothesis we proceed in two stages: 1) identify the “best” model as the model (MST, GEOM, or STRESS) with the most predictions included within our calculated 95% credible intervals for each scaling relationship; and 2) interpret any deviations from the best model by considering how factors specific to savanna systems may interact with the idealizing assumptions of the theoretical model to cause allometric deviations.

## Materials and Methods

### Field Data

We collected data from three savanna sites that span the tropical rainfall gradient in Mali, West Africa. The sites vary in mean annual precipitation, tree architecture, canopy cover and height, fire frequency, grazing intensity, and species composition (Table 2). Across the sites mean annual precipitation ranges from 570–1,400 mm yr^−1^ (north to south) and fire frequency ranges from 0.9 yr^−1^ at the northernmost site (Lakamané) to 0.35 yr^−1^ at the southernmost site (Tiendéga). Large, wild herbivores are effectively absent in West African savannas, but each site does receive some level of grazing by cattle and browsing by goats (Table 2). At each site, we chose ten trees of the dominant species for harvest, except at one site (Tiorola) where we only harvested five individuals.

**Table 2:** Site characteristics.

**Site**	Tiendéga	Tiorola	Lakamané
**Sampled Species**	*Detarium microcarpum*	*Combretum geitynophylum*	*Combretum glutinosum*
**Mean Rainfall (mm y** ^−**1**^ **)**	1,400	1,200	570
**Woody Cover (%)**	60.3	61.3	12.4
**Domestic Animal Density**	Low	Medium	High
**Fire Frequency (y** ^−**1**^ **)**	0.35	0.5	0.9

Woody cover was measured and domestic animal density provisionally estimated in 2008. Fire frequency was extracted from continental-scale data and thus shows broad patterns.

We felled each tree and for every branch with a diameter greater than or equal to 2 cm measured: 1) branch (or basal) diameter within 5 cm of the branch points (or within 10 cm of the soil for basal measurements), 2) length, 3) wood wet weight, and 4) leaf wet weight. We took subsamples of main stem (i.e. trunk) wood (one sample per tree) and leaves (approximately 30 g wet weight per tree) to obtain species-specific dry:wet weight ratios used to account for the contribution of water content to wet weights of wood and leaf. We aggregated biomass data by branch. That is, the biomass (leaf, wood) of each daughter branch was summed for each parent branch to ensure all biomass downstream of any particular branching node is attributed to that branch’s diameter. Since not all trees had branches with diameters greater than 2 cm we only used dry:wet weight ratios from the trunk, but for trees where we took sub-samples of trunk and branch wood there is a near 1∶1 relationship between trunk and branch dry:wet weight ratios (data not shown) indicating there is no systematic variation in dry:wet weight ratios with regards to branch order. The dataset contains observations for 25 individual trees composed of 286 branches (including main stems) representing three savanna tree species: *Deterium microcarpum* Guill. and Perr. (n_tree_ = 10; n_branch = _103), *Combretum geitynophylum* Loefl. (n_tree_ = 5; n_branch = _30), and *Combretum glutinosum* Perr. (n_tree_ = 10; n_branch = _128). We conducted the subsequent analysis using a combined dataset comprised of tree and branch data. Each branch, including the main stem or whole-tree, is treated as an observation and is indexed by tree and species (see *Data analysis: hierarchical Bayesian model*). All data associated with this work is available from Dryad (http://dx.doi.org/10.5061/dryad.4s1d2).

All necessary permits were obtained for the described field studies. All field sites are in public lands administered by the Malian Nature Ministry (Departement des Eaux et Forets). Data collection at field sites was made possible through a memorandum of understanding covering the creation and long-term operation of the sites. Field data collection did not involve or cause harm to any endangered or protected species.

### Scaling Models

The MST botanical model by West, Brown, and Enquist (WBE; [1]) postulates rules that govern plant branching architecture and can serve as a baseline for variation in plant form. In so doing, this model invokes the existence of, and selection for, optimally branching resource distribution networks (e.g. plant vascular systems). In particular, the original WBE model of plant architecture proposes that vascular networks have evolved to minimize hydrodynamic resistance and to maximize the scaling of exchange surfaces such as leaves [1], [20]. Quarter-power scaling then emerges as a consequence of these constraints and physical constraints related to buckling [1]. Based on these assumptions, MST makes specific predictions for the scaling of branch/tree length/height (*l*) and total aboveground biomass (*M*) with branch (or basal) diameter (*D*) (Table 1). Further developments by Enquist and Niklas [21] predict the allocation of total biomass to leaf mass (*L*) and stem mass (*S*) within the plant based on size (Table 1). If ‘space-filling’ and ‘area-preserving’ are the primary evolutionary drivers of network architecture across taxa and resources are homogeneously distributed, then WBE scaling exponents should be identical across divergent taxa that may differ functionally due to other traits [22].

In addition to MST, we also evaluate two other scaling models that invoke biophysical limitations to derive scaling exponents from first principles. As in Price et al. [10], we consider the Stress Similarity model (STRESS) [3] and the Geometric Similarity model (GEOM) [2]. The STRESS model assumes that for a trunk or branch there is a constant maximum biomechanical stress level maintained throughout [3]. This assumption is based on engineering principles of stress levels in beams necessary to resist buckling. GEOM has been considered a null model of plant scaling [10] and it assumes length and radius (or diameter) scale isometrically (i.e., *l 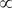
D*, leading to *_bl_*_ = 1_). These models (MST, GEOM, and STRESS) all make predictions assuming an allometrically ideal plant, that is, a plant that follows the assumptions laid out by any given general theory of allometric scaling. An ideal plant is then model-specific. Thus, we refer to any given model prediction as an “ideal” prediction.

We do not explicitly consider the elastic similarity model (ELAS) [3], [23] because the original fractal-branching model of WBE includes the assumption of elastic similarity [1] and thus MST and ELAS make similar predictions for the scaling of mass and length with tree diameter [21], [24]. Also, we do not consider models of increased complexity, such as the PES model described by Price et al. [25] or models that include competitive interactions such as proposed by Muller-Landau et al. [5] or Rüger and Condit [26] because our goal is to focus on simple, universal scaling models that do not need specific environmental data. Specific allometric predictions for all models are in Table 1.

### Data Analysis: Hierarchical Bayesian Model

We used a hierarchical Bayesian (HB) approach to simultaneously estimate multiple scaling relationships using the general allometric power-law in Equation 1 where *Y* is the dependent variable or plant trait/characteristic, *X* is branch (or basal) diameter (hereafter *D* in equations), *a* is a normalizing constant, and *b* is the scaling exponent. Parameters were fit using the log-form of Equation 1:
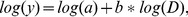
(2)because recent work suggests biological power-laws are best characterized assuming multiplicative error distributions [27], [28].

The hierarchical Bayesian approach allows us to explicitly model measurement error on independent variable *D* and allows for under-represented species to borrow statistical strength by assuming the allometric parameters come from some global population. Moreover, our approach allows us to simultaneously estimate tree, species, and interspecific level scaling parameters using partial pooling [29].

To account for measurement error in diameter (*D*) for each observation *i* we used a Berkson “error-in-variables” model assuming 5% error on at least 5% of trees [30] and used conditioning parameters from Price et al. [10] to inform the prior error distribution, σ_ρ_. We assumed measurement error to be log-normally distributed as:
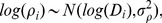
(3)where *ρ_i_* is the latent (“true”) diameter for observation *i* and 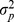 is the measurement error variance. We used a multivariate normal likelihood to estimate the parameters of several scaling relationships simultaneously [10]:
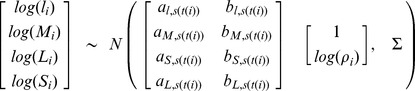
(4)where *a*’s are normalizing constants and *b*’s are scaling exponents for the relationships between *l* (branch length), *M* (total aboveground biomass), *L* (leaf biomass), or *S* (stem biomass) and *ρ*, and Σ is a 4 × 4 covariance matrix. Subscripts *i, t,* and *s* refer to observation, tree, and species respectively and *s*(*t*(*i*)) indicates “species *s* associated with tree *t* associated with observation *i*”.

As suggested by the subscripts, our analysis includes a hierarchical structure to explicitly account for the nested structure of our dataset (i.e. branches nested within individual trees; trees nested within species). Specifically, we account for data dependencies within species and within trees. We account for the fact that all branches within a given tree are related by including a “tree level” in the HB model (denoted by subscript *t*), but we do not account for specific parent-daughter branch relationships. Adding the amount of layers necessary to account for such dependencies in our hierarchical model is unreasonable due to our relatively small sample size. We acknowledge this limitation but we believe the three-level structure described below is sufficiently conservative. Note that “tree level” does not refer to scaling exponents calculated using whole-tree data, but rather the tree level of the HB model.

Scaling exponents for the relationships between *l*, *M*, *L*, and *S* were calculated using the full dataset combining branch and whole-tree data at tree, species, and population levels. Thus, for variable *Y* (*Y = l, M, L,* or *S*) and species *s* associated with tree *t,* the tree-level parameters, *a_s(t)_* and *b_s(t)_*, are hierarchically drawn from species-level parameter distributions with prior:
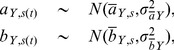
(5)where *a_Y,s_* and *b_Y,s_* are the intraspecific (species-specific) normalizing constants and scaling coefficients, and 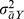 and 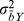 are the within species variances describing tree-to-tree variability in the parameter. To assess the overall tendency of the model coefficients regardless of species but while still explicitly accounting for multiple sources of error (partial pooling) we define 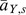 and 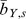 as coming from an overall ‘global’ population [10]:
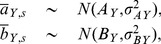
(6)where A and B are the interspecific, population-level normalizing constant and scaling exponent, respectively. The variance terms (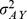 and 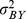) describe the variability among species for both parameters. All priors (for error terms and the hyper-parameters A and B) were chosen to follow non-informative, uniform distributions [31]. We used a non-informative Wishart distribution for the precision matrix (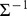) in Equation 4 [10], [29], [32].

We used Markov chain Monte Carlo (MCMC) methods to estimate the joint posterior distributions of each parameter as implemented using JAGS [33] within the statistical package ‘R’ [34]. Three parallel MCMC chains were run with only the covariance matrix Σ initially estimated to define the structure of the matrix. We obtained 1,000,000 iterations of each chain, thinned by 10, after discarding an initial 200,000 iterations as burn-in. We achieved convergence of MCMC chains as assessed using the Heidelberger [35] diagnostic within the ‘coda’ package of ‘R’ [36]. An R script to replicate our analysis is included as supporting information (Supporting Information File S1) as well as JAGS code for the HB model (Supporting Information File S2).

Since our hypothesis is that environmental factors will influence plant allometries we also conducted the analysis above with additional explanatory variables from Table 2. We took two approaches: 1) we included mean annual precipitation, fire frequency, and percent tree cover as potential explanatory variables in Equation 4 (similar to the approach taken by Rüger and Condit [26]), and 2) we included mean annual precipitation, fire frequency, and percent tree cover as hyperparameters in a regression equation that served as a prior for the species-specific normalizing constant (*a_Y,s_*) in Equation 5. However, for both cases the posterior distributions for the coefficients of each variable (except diameter) broadly overlapped zero and *r* ^2^ values did not increase. Likewise, some parameters in our HB model did not achieve convergence with the extra variables included. This is most likely because the environmental variables (specifically mean annual precipitation and fire frequency) in Table 2 are taken from continental-scale, coarse-resolution remote sensing datasets. As such, even though those factors may be important for tree allometries in savannas, the data are not highly resolved enough to be statistically important.

### Data Analysis: Posterior Predictive Checks

To check HB model fit we take a simple approach comparing replicated datasets as simulated from the model to the data that were used to estimate parameters [29]. If the distribution of the simulated data is not congruent with the distribution of the real data then there may be problems with the model itself or with the prior probability distributions [37]. Here we use posterior predictive checks [29] that use a test statistic from the replicated data (*T^rep^*) and an identical test statistic from the real data (*T^obs^*; following the notation of [37]). Using these test statistics we test for lack of fit by calculating *P_B_*, the probability that the replicated data is more extreme than the real data:
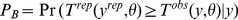
(7)where *θ* is the vector of power-law parameters (*a* and *b*). The model shows lack of fit if *P_B_* is near 0 or 1, since it is a two-tailed probability [38]. Values nearer 0.5 indicate no lack-of-fit. To assess goodness-of-fit we calculate correlation coefficients (*r^2^*) between observed and replicated datasets.

For our log-log regressions we used two test statistics, one to assess the ability of the model to capture the mean tendency of the data (Equation 8), and a second to assess the model's ability to portray the variation in the data (Equation 9). For each trait (length, mass, leaf mass, and stem mass) we used:
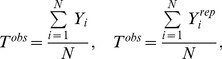
(8)and
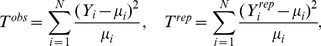
(9)where 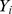 is the real data, 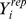 is the replicated data, and 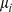 is the model prediction for length, mass, leaf mass, or stem mass. Essentially, Equation 9 uses a sums-of-squares approach to evaluate model fit [37]. We refer to the corresponding 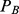 values as 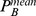 and 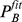 for Equations 8 and 9, respectively.

### Data Analysis: Scaling Model and Exponent Comparison

To compare the scaling models (MST, STRESS, and GEOM) we examined the mean, median, and 95% CIs of the posterior distributions of the global exponents for scaling parameters estimated by our HB model. If a theoretical prediction is included in the 95% CI, then we consider that model supported by the data. More specifically, when the predicted parameters of one of the scaling models fall within the CI of the empirical observations, that model cannot be excluded. We calculate the percentage of all CIs (at all hierarchical levels) that include the theoretical prediction of each model. We consider the scaling model with the highest percentage of inclusion to be the best model.

To compare scaling exponents for a particular relationship among species we examined the overlap of the 95% CIs. Further, we used the HB model to estimate the posterior distribution of the difference between exponents. We then used this distribution to calculate the probability that a difference between two exponents is greater than zero.

## Results

### HB Model Evaluation

**Figure 1:**
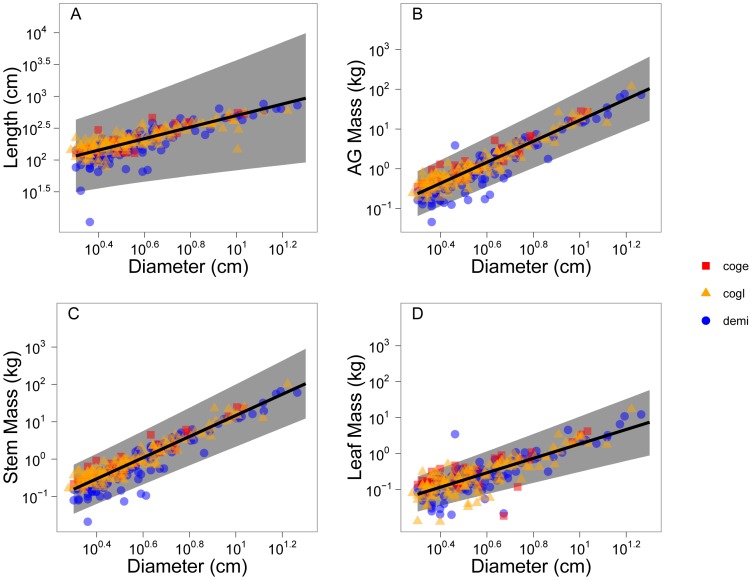
Fitted allometries for each allometric relationship using global level parameters. The symbols correspond to species as according to the legend and are semi-transparent to show overlapping points. Lines show global level (interspecific) mean fit and the shaded regions are the 95% credible intervals. Note that all plots are in log-log space. Species codes: demi, *Detarium microcarpum* (MAP = 1400 mm yr^−1^); coge, *Combretum geitynophylum* (MAP = 1200 mm yr^−1^); cogl, *Combretum glutinosum* (MAP = 570 mm yr^−1^).

All models explain ≥84% of the variation for a given trait (Table 3). Posterior predictive checks show the HB model is capable of replicating data consistent with the mean of the observations, with all 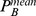 values near 0.5 (Table 3). However, the HB model is less able to accurately replicate the variability inherent in the observed data since all 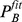 values are nearer to 1 or 0 than 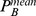 values (Table 3). In particular, when predicting diameter–length scaling there is much unaccounted variability (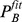 = 0.048). This greater variation in model fit for length and leaf mass scaling compared to aboveground mass and stem mass scaling is also reflected by lower *r^2^* values (Table 3). Raw data and fitted ‘global’ level allometries are shown in Figure 1.

**Table 3:** Posterior predictive checks of the HB model.

Trait	*_r_^2^*	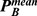	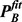
Length	0.85	0.500	0.049
Aboveground mass	0.94	0.502	0.258
Stem mass	0.95	0.501	0.255
Leaf mass	0.85	0.502	0.369

We calculated three test statistics: 1) Pearson’s *r^2^* assessing the correlation between the observed data and the replicated data, 2) 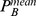 to assess the ability of the model to capture the mean tendency of the data, and 3) 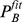 based on a sums-of-squares approach to assess overall model fit including its ability to capture data variability. A value of 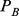 (for both *mean* and *fit*) near 0 or 1 indicates lack of fit; values near 0.5 are acceptable.

### Scaling Exponents: Tree, Species, and ‘Global’ Levels

**Figure 2:**
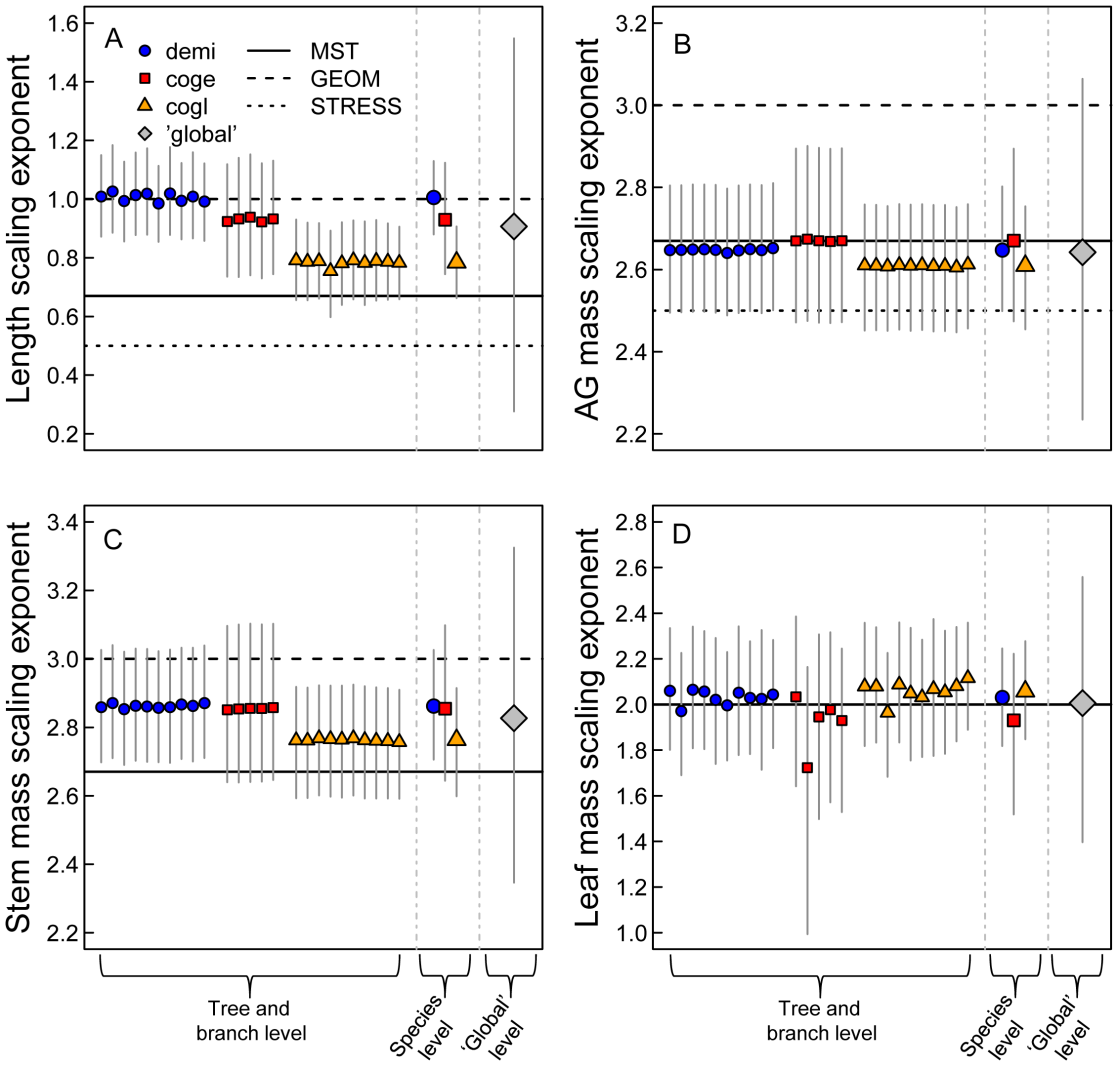
Posterior means and 95% credible intervals of scaling exponents (*b*) at different hierarchical levels. Symbols correspond to the species and the large diamond represents the interspecific, global-level scaling exponent. 95% credible intervals are shown as vertical lines on means. The levels along the x-axis refer to levels in the hierarchical Bayesian model. The horizontal lines represent the theoretical predictions of the three scaling models (note that in D MST and GEOM make the same prediction, see Table 1). Species codes are as in Figure 1. AG mass = aboveground mass.

Within-trees there is considerable branch-level variability as indicated by the 95% CIs associated with tree-level means (Figure 2). Tree-to-tree variability of scaling exponents within species is extremely low for each trait scaling relationship (Table 4 and Figure 2 ‘Tree and branch level’). Only the scaling exponents for leaf mass scaling show substantial tree-to-tree variability (Figure 2D).

**Table 4:** Variance components of the hierarchical Bayesian model for each scaling relationship (diameter vs. trait).

Trait	Tree-level variance ( 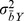 )	Species-level variance( 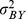 )
Length	0.0016 (1.71e-6, 0.007)	0.2700 (0.002, 2.281)
Abovegroundmass	0.0003 (2.77e-7, 0.002)	0.1323 (1.83e-5, 1.385)
Stem mass	0.0005 (5.29e-7, 0.003)	0.1698 (3.99e-5, 1.676)
Leaf mass	0.0160 (3.18e-5, 0.067)	0.2160 (3.56e-5, 2.026)

Means are shown with 95% credible intervals displayed in parentheses.

**Figure 3:**
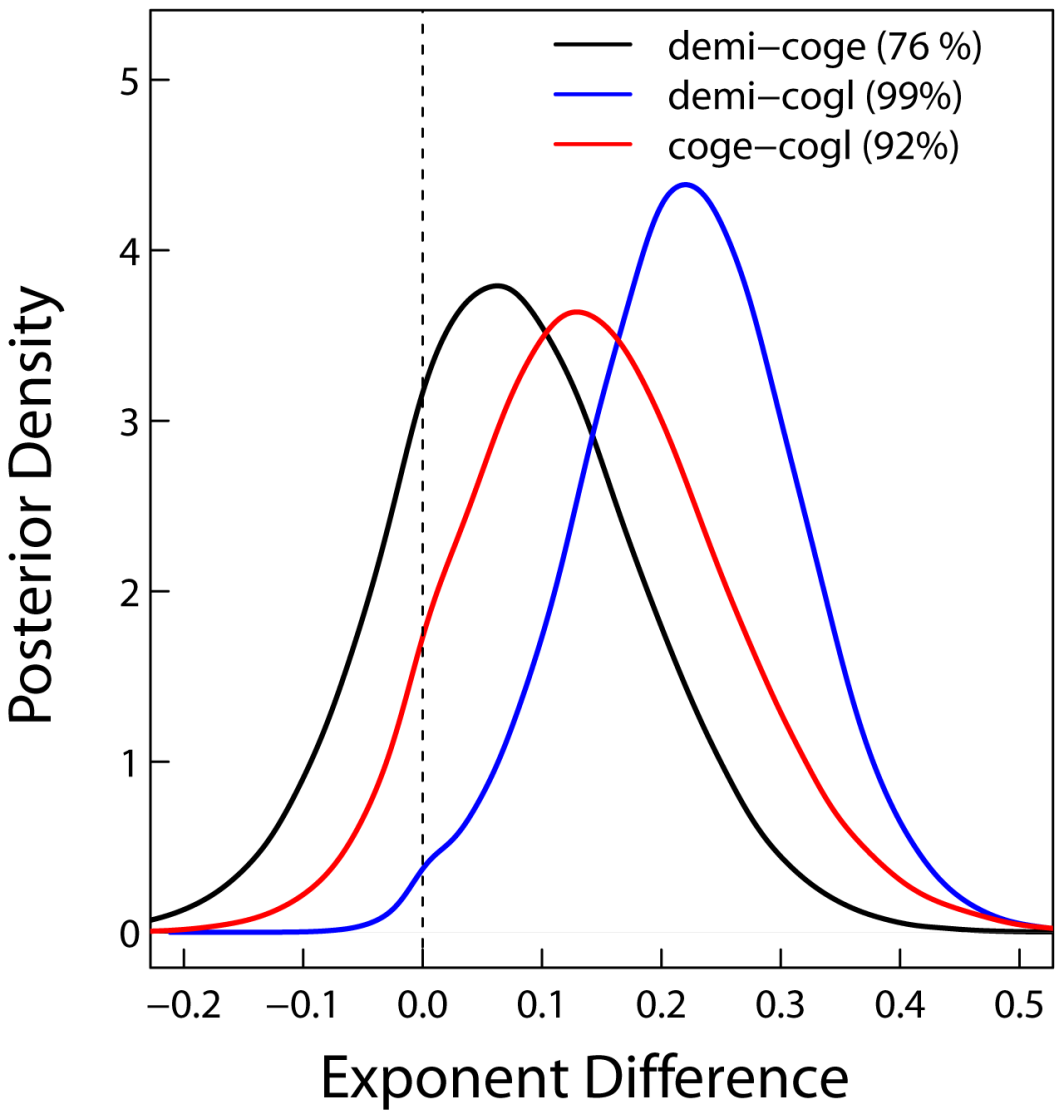
Posterior densities of the difference between species-specific scaling exponents. The dashed line shows a difference of zero. Species contrasts are indicated by color as in the legend where “demi–coge” means the scaling exponent of *D. microcarpum* minus the scaling exponent of *C. geitynophylum*. The probability that a specific difference is greater than zero (which can be considered a significant difference between exponents) is displayed in parentheses in the legend. Species codes are as in Figure 1.

**Figure 4:**
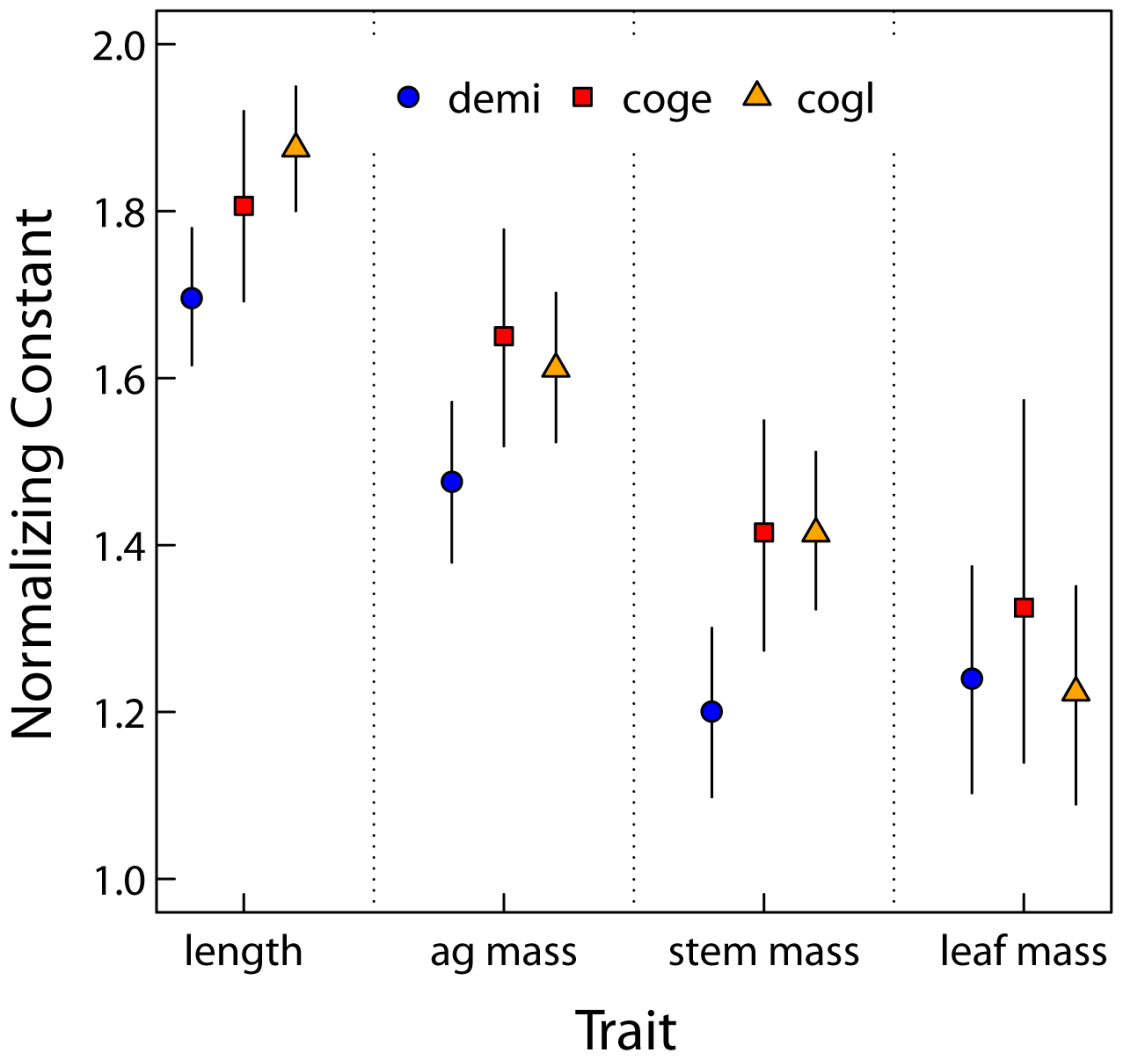
Species level posterior means and 95% credible intervals of normalization constants (*a*). Species codes are as in Figure 1 and “Traits” along x-axis refer to the scaling of diameter with that trait (e.g., “length” refers to the normalizing constants for the scaling relationship *l* = *aD^b^*). Symbols correspond to the species and 95% credible intervals are shown as vertical lines on means.

At the species level, only length scaling exponents show any interspecific variability. Fitted length scaling exponents are greater than predicted by MST and increase with mean annual precipitation (Figure 2A, ‘Species level’ and Table 2). Importantly, for the species on the extreme ends of the savanna gradient we sampled (*D. microcarpum* and *C. glutinosum*), there is a 99% probability that the difference between length scaling exponents is greater than zero (Figure 3). There is 76% probability the scaling exponents for *D. microcarpum* and *C. geitynophylum* are different, and a 92% probability the scaling exponents for *C. geitynophylum* and *C. glutinosum* are different (Figure 3). Similarly, the length-scaling normalization constants also show a directional trend, but with *D. microcarpum* having the lowest value and *C. glutinosum* having the highest value (Figure 4).

Given the low variance at the tree-level, species-specific exponents have 95% CIs that are primarily driven by branch-level variance, not tree-level variance. For example, for all trait scaling relationships and all species the average difference in CI width between the tree level and the species level is 0.037. However, species-level variance is greater than tree-level variance (Table 4). Except for the leaf mass scaling relationship, the normalization constants of *D. microcarpum* are lower than that of *C. geitynophylum* and *C. glutinosum* (Figure 4) and the 95% CIs do not overlap the means.

The combination of branch-level variability (95% CIs on tree means) and variability among species results in wide 95% CIs at the ‘global’, interspecific level (Figure 1, 2). Tree-level variability does not contribute greatly to interspecific variation since that variance is low (Table 4). The ‘global’ means and associated 95% CIs indicate the overall distribution from which subsequent levels (tree and species) are derived. These distributions serve as indicators of ‘naturally possible’ scaling exponents regardless of species.

The scaling exponents arising from our dataset are generally consistent with those calculated in other studies using a diversity of species and tree functional types. For example, diameter-length scaling exponents tend to fall between values of approximately 0.3–0.8 [5], [9], [10], [32] and diameter-aboveground biomass scaling exponents tend to fall between approximately 2.0–3.0 [5], [10], [20]. Few studies have examined the scaling of diameter to stem or leaf biomass specifically. But, our results for stem and leaf biomass scaling are consistent with those presented by Enquist and Niklas [21] in their initial derivation of the proposed MST exponents. Likewise, our results for leaf mass scaling are broadly consistent with those presented by Price et al. [10], using 2,362 individuals from over 100 species, that show leaf area scaling exponents (which are equivalent to leaf biomass exponents under the assumption that leaf biomass and area scales isometrically) to be in the range of approximately 1.3–2.8.

### Scaling Exponents: Empirical Support for Theoretical Scaling Models

The wide 95% CIs at the ‘global’, interspecific level precludes the exclusion of any of the theoretical scaling models. However, the universal models we evaluated make predictions assuming species-specific normalizing constants that influence the scaling exponents [22]. As such, it is important to evaluate the models with reference to all the levels in our HB model (the tree and species levels). At the tree and species levels, GEOM is most supported for length scaling and MST under predicts the length scaling exponents (Figure 2A). MST is generally supported for aboveground mass scaling with STRESS receiving nominal support (Figure 2B). MST and GEOM make predictions for biomass partitioning and they perform reasonably well but with MST tending to under-predict and GEOM tending to over-predict wood-mass scaling (Figure 2C). For leaf-mass scaling MST and GEOM are equally well supported (since the predictions are identical) given their abilities to capture the means (Figure 2D). For all scaling relationships, and including all hierarchical levels (29 calculated exponents per scaling relationship), MST predictions are included in 75% of the credible intervals and GEOM predictions in 57%. For the two scaling relationships that all three models predict (length and aboveground mass) MST predictions are included in 70% of CIs, GEOM in 33%, and STRESS in 52%.

## Discussion

Departures from the ideal predictions of scaling models that do not include environmental factors and variability are to be expected in natural settings where local conditions may select for modified allometries. As such, our goal was to evaluate the extent to which variable environments result in departures of tree allometries from ideal predictions. The ability to determine appropriate allometric relationships in trees is critical to scaling carbon and water fluxes from the leaf to the ecosystem level. Since there is an urgent need to better understand terrestrial dynamics of West African savanna ecosystems in light of current land use change [39] and future climate change [40], here we calculated scaling exponents and tested the utility of popular allometric scaling models in these systems. Since savannas have variable rainfall, fire, and herbivory regimes, we aimed to determine if theoretical models of plant form based in metabolic and mechanical scaling models could successfully be used in these ecosystems to scale allometries.

### Allometric Convergence among and within Savanna Trees

Despite differences in bottom-up (mean annual precipitation, light availability) and top-down (fire, herbivory) forces important to savanna trees [17], [41]–[43], tree and branch scaling from three species from three sites appear to converge on similar mean allometries describing stem length, total above ground biomass and the stem and leaf mass components of total aboveground biomass (Figure 2, 4). Thus, it appears that scaling characteristics in savanna trees converge on mean relationships among and within trees, indicating that some set of universal scaling rules applies. While the mean scaling exponents overlap among trees, there are different amounts of variation associated with exponents at each hierarchical level as discussed below.

Across all species, the most variability in exponent estimations exists around the tree and branch level scaling exponents (Figure 2, ‘Tree and branch levels’ 95% CIs). This indicates that branches may have a greater “scaling space” [44] than trees due to different limitations on mechanical strength and resource transport related to network size. For example, saplings tend to violate the MST assumption of a space-filling branching, and thus elastic similarity [3], [45], and there is evidence branches violate this assumption as well [46]. Likewise, it is important to note that had we included branches smaller than 2 cm in diameter our exponent estimates may have been more variable. von Allmen et al. [46] show that branches where diameter (*D*) is less than 2 cm tend to violate the elastic similarity assumption. As such, our results are biased toward branches and stems that meet the elastic similarity assumption.

With regard to exponents, the greatest variation among and within individual trees occurs in the leaf mass scaling exponents (Figure 2D, ‘Tree and branch levels’). It is well known that leaf area and biomass are variable in space and time at a variety of scales [47]–[49]. As such, leaf biomass may be more plastic in response to micro-environmental conditions than other ‘wood-based’ traits (aboveground and stem mass and length) that are more tightly linked to diameter through mechanical constraints [3] and metabolic efficiency [1]. Discovering how micro-environmental conditions and tree size interact to produce tree- and branch-specific allometric relationships, and the width of allowable allometries, is an important avenue for future research.

### Empirical Support for Theoretical Scaling Models

Previous tests of ‘universal’ scaling models of plant form and function have found only limited empirical support for the theoretical models considered here [5], [9], [10], [26], [32]. Our analysis shows that none of the models tested (MST, GEOM, and STRESS) can be definitively excluded at the ‘global’, interspecific level (Figure 2), though the models do differ in overall performance at the species and tree levels as also found in a comprehensive analysis by Price et al. [10]. This is particularly interesting given the broad climate and disturbance gradient from which the data were gathered, the diversity of species considered (Table 2), and the fact that we tested these models using branch-level data. In aggregate, however, MST outperforms STRESS and GEOM in predicting the scaling relationships we observed across all levels (Figure 2). Many other studies also report support for the predictions and assumptions related to external branching architecture as defined by WBE and MST. For example, area-preserving branching has been widely reported [3], [50]–[59], and recent studies find empirical support for elastic similarity [46] and self-similarity (Bentley et al. unpublished data). However, since the length-scaling exponents are most consistent with the predictions of GEOM (Figure 2), it will be important for future research to focus on the underlying assumptions of the competing models [60]. Only then can we truly identify departures from model predictions, as opposed to comparing data to an incorrect or incomplete model.

It is important to note that we did not consider models of differing complexity. Several authors have begun to relax MST assumptions [25] or include competitive interactions [5], [26] to better account for diversity in botanical form and function [61]. These more complex models have been shown to provide better fits to empirical data [10]. Though we did not evaluate such models here, since our focus was on strictly ‘universal’ models and we did not have adequate data, it is likely they would outperform the models we did evaluate. Nonetheless, our analysis does indicate MST may best capture the underlying constraints on allometric relationships, so extensions of it may prove most useful [25], [62].

### Implications for MST in Variable Ecosystems

Given the data in hand and the competing scaling theories we tested, we consider MST the ‘best’ model since its predictions were included in the greatest percentage of credible intervals (see *Results*). If MST is considered the ‘best’ model, what can we conclude regarding deviations from MST predictions?

The most striking deviation from MST predictions occurs in the scaling of diameter to length (Figure 2A) where MST shows a strong trend for under predicting the scaling exponent. In concert with this deviation from the MST prediction, the length-scaling parameters display a clear directional trend among species: the exponents increase with mean annual precipitation and tree cover (Table 2, Figure 2A, 3) while the normalization constants decrease (Figure 4). The normalization constants absorb some of the environmental variation among sites, as predicted by MST [20] and reflected in our analysis (Figure 4), but not enough to produce convergent exponents. We consider this variation among species’ scaling exponents to represent an ecologically important deviation from MST and hypothesize that the following biological processes may differentially influence the scaling of tree height and branch length in savannas at multiple levels: 1) long-term adaptation to fire in savanna trees, and 2) differences in the intensity of resource competition among sites. Environmental factors such as fire frequency, woody cover (proxy for light competition), and mean annual precipitation were not statistically important in our model, but, as discussed previously, this was likely due to the spatial resolution of the environmental data as opposed to their lack of importance.

Fire plays a critical role in regulating savanna structure by constraining recruitment of juvenile trees into adult classes [43]. As such, fire is a strong selective force in savannas [63]–[67], and trees in fire-prone ecosystems may benefit from rapid vertical growth to escape the fire zone [19], [68], [69]. Therefore, we would expect, and our data shows, length-scaling exponents to trend toward values greater than expected from MST. As has been suggested before [19], [68], [70], we hypothesize that savanna trees have evolved, via natural selection, to allocate growth toward height or branch length at the expense of mechanical stability and optimization of resource transport. Fire is a selective force in savannas that overrides the first-order optimization of plant vascular networks in response to physical (water and nutrient transport) and mechanical (buckling) constraints. All species in our dataset reflect the allometric influence of fire toward a greater exponent, even while showing some interspecific variability (Figure 2A). Trees in South African and Brazilian savannas have shown qualitatively and quantitatively similar allometric trends [9], [19], [71], suggesting a universal trade-off in savannas between fire escape and mechanical stability and optimization of resource transport.

While fire can explain overall deviation from the MST prediction, multiple selective pressures related to resource competition may be operating simultaneously at the intraspecific level (Figure 2A, 4). For example, light has been shown to influence forest tree allometries [5] and predictions based on optimal partitioning theory indicate that plants in reduced sunlight shift allocation toward height to gain a competitive advantage for light capture [72]. Since our calculated intraspecific exponents for length scaling increased with precipitation and woody cover (Table 2, Figure 2A, 3), our results are consistent with this theory. In dense savannas, as in forests, the competition for light may select for modified allometries with scaling exponents for diameter vs. height/length greater than 0.67 as observed here (Figure 1A). However, light competition in savannas has received very little attention, and water may still be the limiting factor. In that case we would not expect a light response in allometries.

The directional trend observed among species could also be explained by an interaction between a bark thickness–height growth tradeoff and access to resources. Work in African, Australian, and Brazilian savannas suggests top-kill/mortality of savanna trees due to fire is most correlated (negatively) with bark thickness [69], [70], [73]. Thus, Lawes et al. [73] argue that fire escape height is better conceived as the height required to attain bark thick enough to resist fire damage; as opposed to simply being tall enough to avoid branch inflammation. As such, trees in fire-prone savannas must invest biomass in bark growth at the expense of height growth [73]. It follows, then, that this trade-off may be more pronounced in arid savannas where moisture is more limiting to overall growth. Then, as observed in this study, the fire response in arid savannas would lead to lower length-scaling exponents than in more mesic savannas (Figure 2A). This proposed interaction among height (or length), bark thickness, and resource availability has yet to be thoroughly investigated (but see [69]).

In addition to light availability and fire frequency, browsing can also lead to intra and interspecific variation of length scaling and has been shown to influence savanna tree architecture [9], [19], [42]. Our dataset did not come from sites with large browsers, but interestingly, length-scaling exponents calculated for South African savanna trees protected from and exposed to large browsers (e.g., giraffe) are remarkably similar to our estimates (in the range of 0.57–0.74 [9]). Moncrieff et al. [9] do show that browsing can decrease length-scaling exponents below both our calculated value and the MST predicted value. However, the deviations from MST observed by Moncrieff et al. [9] on trees subject to browsing may reflect near-term physiological responses to mechanical damage rather than long-term adaptations in growth strategy as proposed here.

### Conclusions

Ultimately, observed plant allometries in any system will reflect some combination of multiple trade-offs that may be difficult to capture in general theories of plant form and function, such as MST. Deviations from the predictions of MST make intuitive sense when we consider the multiple costs, benefits and selective forces active in savannas. While plant architecture may reflect, in part, the morphological adaptations that optimize the efficiency of resource transport, when subject to selective forces unrelated to transport (e.g. mortality of shorter individuals in fire, or competition with neighbors for light) we can anticipate selection of traits (e.g. longer branch node-lengths) that balance the benefits of ‘escape’ from fire and competition with the potential mechanical and transport ‘costs’ associated with longer and thinner branches. However, unlike Moncrieff et al. [9] who conclude that general theories including MST may be “neither general nor predictive in systems with frequent disturbance”, we find that, even in disturbance-prone savannas, MST is generally consistent with observations (i.e. allometries for leaf, stem, and total mass). Further, in those situations where observations are inconsistent with MST (i.e. stem length) we find that departure from theory corresponds with expected tradeoffs related to disturbance and competitive interactions. Thus, we suggest two future research priorities: 1) detailed studies that empirically test the validity of model assumptions related to length scaling and 2) theoretical work aimed toward quantitatively predicting the magnitude and direction of allometric modifications in response to selective drivers other than core physical principles. In combination, such work could lead to an improved plant scaling model that best represents observed scaling relationships in variable ecosystems.

## Supporting Information

Information S1 **R script to set-up and call the hierarchical Bayesian model as specified in the JAGS code in File S2.**
(TXT)

Information S2 **JAGS code for hierarchical Bayesian model.**
(TXT)

## References

[1] West GB, Brown JH, Enquist BJ (1999) A general model for the structure and allometry of plant vascular systems. Nature 400: 664–667.

[2] Niklas KJ (1994) Plant allometry the scaling of form and process. Chicago: University of Chicago Press.

[3] McMahon TA, Kronauer RE (1976) Tree structures: Deducing the principle of mechanical design. Journal of Theoretical Biology 59: 443–466. 957700 10.1016/0022-5193(76)90182-x

[4] Kozĺowski J, Konarzewski M (2004) Is West, Brown and Enquist’s model of allometric scaling mathematically correct and biologically relevant? Functional Ecology 18: 283–289.

[5] Muller-Landau HC, Condit RS, Chave J, Thomas SC, Bohlman SA, et al (2006) Testing metabolic ecology theory for allometric scaling of tree size, growth and mortality in tropical forests. Ecology Letters 9: 575–588. 16643303 10.1111/j.1461-0248.2006.00904.x

[6] Tilman D, HilleRisLambers J, Harpole S, Dybzinski R, Fargione J, et al (2004) Does metabolic theory apply to community ecology? It’s a matter of scale. Ecology 85: 1797–1799.

[7] Coomes DA (2006) Challenges to the generality of WBE theory. Trends in Ecology & Evolution 21: 593–596. 16987565 10.1016/j.tree.2006.09.002

[8] Coomes DA, Allen RB (2009) Testing the Metabolic Scaling Theory of tree growth. Journal of Ecology 97: 1369–1373.

[9] Moncrieff GR, Chamaillé-Jammes S, Higgins SI, O’Hara RB, Bond WJ (2011) Tree allometries reflect a lifetime of herbivory in an African savanna. Ecology 92: 2310–2315. 22352170 10.1890/11-0230.1

[10] Price CA, Ogle K, White EP, Weitz JS (2009) Evaluating scaling models in biology using hierarchical Bayesian approaches. Ecology Letters 12: 641–651. 19453621 10.1111/j.1461-0248.2009.01316.x PMC2730548

[11] Brown JH, West GB, Enquist BJ (2005) Yes, West, Brown and Enquist’s model of allometric scaling is both mathematically correct and biologically relevant. Functional Ecology 19: 735–738.

[12] Stark SC, Bentley LP, Enquist BJ (2010) FORUM: Response to Coomes & Allen (2009)‘Testing the metabolic scaling theory of tree growth’. Journal of Ecology 99: 741–747.

[13] Ellis JE, Swift DM (1988) Stability of African Pastoral Ecosystems: Alternate Paradigms and Implications for Development. Journal of Range Management 41: 450–459.

[14] Sankaran M, Hanan NP, Scholes RJ, Ratnam J, Augustine DJ, et al (2005) Determinants of woody cover in African savannas. Nature 438: 846–849. 16341012 10.1038/nature04070

[15] Chesson P, Gebauer RLE, Schwinning S, Huntly N, Wiegand K, et al (2004) Resource pulses, species interactions, and diversity maintenance in arid and semi-arid environments. Oecologia 141: 236–253. 15069635 10.1007/s00442-004-1551-1

[16] Russo SE, Wiser SK, Coomes DA (2007) Growth–size scaling relationships of woody plant species differ from predictions of the Metabolic Ecology Model. Ecology Letters 10: 889–901. 17845289 10.1111/j.1461-0248.2007.01079.x

[17] Sankaran M, Ratnam J, Hanan NP (2004) Tree–grass coexistence in savannas revisited – insights from an examination of assumptions and mechanisms invoked in existing models. Ecology Letters 7: 480–490.

[18] Ratnam J, Bond WJ, Fensham RJ, Hoffmann WA, Archibald S, et al (2011) When is a ‘forest’ a savanna, and why does it matter? Global Ecology and Biogeography 20: 653–660.

[19] Archibald S, Bond WJ (2003) Growing tall vs growing wide: tree architecture and allometry of Acacia karroo in forest, savanna, and arid environments. Oikos 102: 3–14.

[20] Enquist BJ (2002) Universal scaling in tree and vascular plant allometry: toward a general quantitative theory linking plant form and function from cells to ecosystems. Tree Physiology 22: 1045–1064. 12414366 10.1093/treephys/22.15-16.1045

[21] Enquist BJ, Niklas KJ (2002) Global Allocation Rules for Patterns of Biomass Partitioning in Seed Plants. Science 295: 1517–1520. 11859193 10.1126/science.1066360

[22] Enquist BJ, Bentley LP (2012) Land Plants: New Theoretical Directions and Empirical Prospects. In: Sibly R, Brown JH, Kodric-Brown A, editors. Metabolic Ecology: A Scaling Approach. Hoboken, NJ: Wiley-Blackwell. 164–187.

[23] McMahon T (1973) Size and Shape in Biology. Science 179: 1201–1204. 4689015 10.1126/science.179.4079.1201

[24] Price CA, Gilooly JF, Allen AP, Weitz JS, Niklas KJ (2010) The metabolic theory of ecology: prospects and challenges for plant biology. New Phytologist 188: 696–710. 20819176 10.1111/j.1469-8137.2010.03442.x

[25] Price CA, Enquist BJ, Savage VM (2007) A general model for allometric covariation in botanical form and function. Proceedings of the National Academy of Sciences 104: 13204–13209. 10.1073/pnas.0702242104 PMC1941814 17664421

[26] Rüger N, Condit R (2012) Testing metabolic theory with models of tree growth that include light competition. Functional Ecology 26: 759–765.

[27] Xiao X, White EP, Hooten MB, Durham SL (2011) On the use of log-transformation vs. nonlinear regression for analyzing biological power laws. Ecology 92: 1887–1894. 22073779 10.1890/11-0538.1

[28] Kerkhoff AJ, Enquist BJ (2009) Multiplicative by nature: Why logarithmic transformation is necessary in allometry. Journal of Theoretical Biology 257: 519–521.

[29] Gelman A, Hill J (2009) Data analysis using regression and multilevel/hierarchical modeling. Cambridge, UK: Cambridge University Press.

[30] Dellaportas P, Stephens DA (1995) Bayesian Analysis of Errors-in-Variables Regression Models. Biometrics 51: 1085–1095.

[31] Gelman A (2006) Prior distributions for variance parameters in hierarchical models. Bayesian Analysis 1: 515–533.

[32] Dietze MC, Wolosin MS, Clark JS (2008) Capturing diversity and interspecific variability in allometries: A hierarchical approach. Forest Ecology and Management 256: 1939–1948.

[33] Plummer M (2003) JAGS: a program for analysis of Bayesian graphical models using Gibbs sampling. In: Hornik K, Leish F, Zeileis A, editors. Proceedings of the 3rd International Workshop on Distributed Statistical Computing. Vienna, Austria.

[34] Team RDC (2012) R: a language environment for statistical computing. R Foundation for Statistical Computing, Vienna, Austria.

[35] Heidelberger P, Welch PD (1983) Simulation run length control in the presence of an initial transient. Operations Research 31: 1109–1144.

[36] Plummer M, Best N, Cowles K, Vines K (2010) coda: Output analysis and diagnostics for MCMC. R package version 0.14–4. R package version 0.14–4 ed: http://CRAN. R-project. org/package = coda.

[37] Hobbs NT, Andrén H, Persson J, Aronsson M, Chapron G (2012) Native predators reduce harvest of reindeer by Sámi pastoralists. Ecological Applications 22: 1640–1654. 22908719 10.1890/11-1309.1

[38] Gelman A, Carlin JB, Stern HS, Rubin DB (2004) Bayesian data analysis. London: Chapman and Hall/CRC.

[39] Anyamba A, Tucker CJ (2005) Analysis of Sahelian vegetation dynamics using NOAA-AVHRR NDVI data from 1981–2003. Journal of Arid Environments 63: 596–614.

[40] Held IM, Delworth TL, Lu J, Findell KL, Knutson TR (2005) Simulation of Sahel drought in the 20th and 21st centuries. Proceedings of the National Academy of Sciences of the United States of America 102: 17891–17896. 16322101 10.1073/pnas.0509057102 PMC1312412

[41] Hoffmann WA, Geiger EL, Gotsch SG, Rossatto DR, Silva LCR, et al (2012) Ecological thresholds at the savanna-forest boundary: how plant traits, resources and fire govern the distribution of tropical biomes. Ecology Letters 15: 759–768. 22554474 10.1111/j.1461-0248.2012.01789.x

[42] Staver AC, Bond WJ, Cramer MD, Wakeling JL (2012) Top-down determinants of niche structure and adaptation among African Acacias. Ecology Letters 15: 673–679. 22507561 10.1111/j.1461-0248.2012.01784.x

[43] Hanan NP, Sea WB, Dangelmayr G, Govender N (2008) Do fires in savannas consume woody biomass? A comment on approaches to modeling savanna dynamics. American Naturalist 171: 851–856. 10.1086/587527 18462133

[44] Sperry JS, Smith DD, Savage VM, Enquist BJ, McCulloh KA, et al (2012) A species-level model for metabolic scaling in trees I. Exploring boundaries to scaling space within and across species. Functional Ecology 26: 1054–1065.

[45] Niklas KJ, Spatz H-C (2004) Growth and hydraulic (not mechanical) constraints govern the scaling of tree height and mass. Proceedings of the National Academy of Sciences of the United States of America 101: 15661–15663. 15505224 10.1073/pnas.0405857101 PMC524850

[46] von Allmen EI, Sperry JS, Smith DD, Savage VM, Enquist BJ, et al (2012) A species-level model for metabolic scaling of trees II. Testing in a ring- and diffuse-porous species. Functional Ecology 26: 1066–1076.

[47] Myneni RB, Yang W, Nemani RR, Huete AR, Dickinson RE, et al (2007) Large seasonal swings in leaf area of Amazon rainforests. Proceedings of the National Academy of Sciences 104: 4820–4823. 10.1073/pnas.0611338104 PMC1820882 17360360

[48] Sultan SE (2000) Phenotypic plasticity for plant development, function and life history. Trends in Plant Science 5: 537–542. 11120476 10.1016/s1360-1385(00)01797-0

[49] Osada N, Takeda H, Furukawa A, Awang M (2001) Leaf dynamics and maintenance of tree crowns in a Malaysian rain forest stand. Journal of Ecology 89: 774–782.

[50] Barker SB, Cumming G, Horsfield K (1973) Quantitative morphometry of the branching structure of trees. Journal of Theoretical Biology 40: 33–43. 4723553 10.1016/0022-5193(73)90163-x

[51] Bertram JA (1989) Size-dependent differential scaling in branches: the mechanical design of trees revisited. Trees 3: 241–253.

[52] Costes E, Guédon Y (2012) Deciphering the ontogeny of a sympodial tree. Trees 26: 865–879.

[53] Dahle G, Grabosky J (2010) Allometric patterns in Acer platanoides (Aceraceae) branches. Trees 24: 321–326.

[54] Day JS, Gould KS (1997) Vegetative Architecture of Elaeocarpus hookerianus. Periodic Growth Patterns in Divaricating Juveniles. Annals of Botany 79: 607–616.

[55] Horn HS (2000) Twigs, trees, and the dynamics of carbon in the landscape. In: Brown JH, West GB, editors. Scaling in Biology. Oxford: Oxford University Press.

[56] Leopold LB (1971) Trees and streams: The efficiency of branching patterns. Journal of Theoretical Biology 31: 339–354. 5557082 10.1016/0022-5193(71)90192-5

[57] Renton M, Guédon Y, Godin C, Costes E (2006) Similarities and gradients in growth unit branching patterns during ontogeny in ‘Fuji’ apple trees: a stochastic approach. Journal of Experimental Botany 57: 3131–3143. 16936222 10.1093/jxb/erl075

[58] Shinozaki K, Yoda K, Hozumi K, Kira T (1964) A quantitative analysis of plant form;the pipe model theory,1. Japanese Journal of Ecology 14: 97–105.

[59] Sone K, Noguchi K, Terashima I (2005) Dependency of branch diameter growth in young Acer trees on light availability and shoot elongation. Tree Physiology 25: 39–48. 15519984 10.1093/treephys/25.1.39

[60] Price CA, Weitz JS, Savage VM, Stegen J, Clarke A, et al. (2012) Testing the metabolic theory of ecology. Ecology Letters: 1465–1474. 10.1111/j.1461-0248.2012.01860.x 22931542

[61] Price CA, Weitz JS (2012) Allometric covariation: a hallmark behavior of plants and leaves. New Phytologist 193: 882–889. 22403825 10.1111/j.1469-8137.2011.04022.x

[62] Price CA, Enquist BJ (2007) Scaling mass and morphology in leaves: an extension of the WBE model. Ecology 88: 1132–1141. 17536400 10.1890/06-1158

[63] Bond WJ, Woodward FI, Midgley GF (2005) The global distribution of ecosystems in a world without fire. New Phytologist 165: 525–538. 15720663 10.1111/j.1469-8137.2004.01252.x

[64] Bond WJ, Keeley JE (2005) Fire as a global ‘herbivore’: the ecology and evolution of flammable ecosystems. Trends in Ecology & Evolution 20: 387–394. 16701401 10.1016/j.tree.2005.04.025

[65] Bond WJ (2008) What Limits Trees in C4 Grasslands and Savannas? Annual Review of Ecology, Evolution, and Systematics 39: 641–659.

[66] Staver AC, Archibald S, Levin S (2011) Tree cover in sub-Saharan Africa: Rainfall and fire constrain forest and savanna as alternative stable states. Ecology 92: 1063–1072. 21661567 10.1890/10-1684.1

[67] Staver AC, Archibald S, Levin SA (2011) The global extent and determinants of savanna and forest as alternative biome states. Science 334: 230–232. 21998389 10.1126/science.1210465

[68] Hoffmann WA, Orthen B, Nascimento PKVD (2003) Comparative Fire Ecology of Tropical Savanna and Forest Trees. Functional Ecology 17: 720–726.

[69] Gignoux J, Clobert J, Menaut J-C (1997) Alternative fire resistance strategies in savanna trees. Oecologia 110: 576–583. 28307253 10.1007/s004420050198

[70] Hoffmann WA, Adasme R, Haridasan M, T. de Carvalho M, Geiger EL, et al (2009) Tree topkill, not mortality, governs the dynamics of savanna–forest boundaries under frequent fire in central Brazil. Ecology 90: 1326–1337. 19537552 10.1890/08-0741.1

[71] Dodonov P, Lucena IC, Leite MB, Silva Matos DM (2011) Allometry of some woody plant species in a Brazilian savanna after two years of a dry season fire. Brazilian Journal of Biology 71: 527–535. 10.1590/s1519-69842011000300025 21755173

[72] McConnaughay KDM, Coleman JS (1999) Biomass allocation in plants: ontogeny or optimality? A test along three resource gradients. Ecology 80: 2581–2593.

[73] Lawes MJ, Adie H, Russell-Smith J, Murphy B, Midgley JJ (2011) How do small savanna trees avoid stem mortality by fire? The roles of stem diameter, height and bark thickness. Ecosphere 2: art42.

